# Assessing stress physiology within a conservation breeding program for an endangered species

**DOI:** 10.1093/conphys/coad041

**Published:** 2023-06-09

**Authors:** S Falconer, M McAdie, G Mastromonaco, A I Schulte-Hostedde

**Affiliations:** Department of Biology/School of Natural Sciences, Laurentian University, 935 Ramsey Lake Rd, Sudbury, Canada, ON P3E 2C6; Marmot Recovery Foundation, PO Box 2332 Stn A, Nanaimo, BC, Canada, V9R 6X6; Reproductive Sciences Unit, Toronto Zoo, 361A Old Finch Avenue, Scarborough, Ontario, Canada, M1B 5K7; Department of Biology/School of Natural Sciences, Laurentian University, S-614, Science Building, 935, Ramsey Lake Rd, Sudbury, Canada, ON P3E 2C6

**Keywords:** Vancouver Island marmot, neutrophil, Lymphocyte

## Abstract

Conservation breeding programs typically involve the management of individuals both *in* and *ex situ*, so it is vital to understand how the physiology of managed species changes in these environments to maximize program outcomes. The Vancouver Island marmot (VIM; *Marmota vancouverensis*) is one species that has been managed in a conservation breeding program to recover the critically low wild population. Previous research has shown there are differences in hair glucocorticoid concentrations for VIMs in different managed groups in the program. Therefore, we used >1000 blood samples collected since the program’s inception to assess the neutrophil to lymphocyte (N:L) ratio among captive, pre-release, post-release and wild populations as another metric of stress. *In situ* VIM populations were found to have a significantly higher N:L ratio than *ex situ* populations, suggesting that the wild is a more physiologically challenging environment than managed care. Moreover, the effect of age, sex and the month of sampling on the N:L ratio were found to be different for each population. Age had the greatest magnitude of effect in the wild population, and sex was only significant in *ex situ* populations. This study provided previously unknown insights into the physiology of VIMs and increased post-release monitoring will be useful in the future to fully understand how physiology may be contributing to differences in survival of VIMs in the program.

## Teaser

We used blood samples from the Vancouver Island marmot recovery program to compare the neutrophil to lymphocyte (N:L) ratio among managed groups. We did find differences in this metric between groups as well as differences in what is contributing to variation within groups.

## Introduction

Conservation initiatives must use scientific evidence to develop management strategies and to monitor program success. Conservation physiology is useful because it provides evidence of cause and effect for the management of programs (Wikelski and Cooke, 2006; Cooke *et al.,* 2013). Conservation physiology has been applied in a variety of biological contexts including evaluating anthropogenic disturbances and ecosystem management, yet there are further opportunities for its application (Cooke *et al.,* 2013, 2020). A key area for conservation physiology is in conservation breeding programs, especially those that involve the release of captive bred animals (Cooke *et al.,* 2020). Because these programs involve the management of species both *in* and *ex situ*, understanding changes in the physiology of managed animals can ultimately increase the program’s effectiveness.

Stress physiology is an area that may be of particular interest to the management of conservation breeding programs. For vertebrates, a stress response is coordinated by the hypothalamus—pituitary—adrenal (HPA) axis, where stimulation of this axis ultimately results in the secretion of glucocorticoids (GCs) whose diverse effects on the body allow the individual to cope with the stressor (Denver, 2009). Although the secretion of GCs is part of normal functioning, prolonged secretion (known as chronic stress) can have negative consequences on the individual (Johnstone *et al.,* 2012; Lattin and Kelly, 2020). In an effort to increase transparency and circumvent possible miscommunications of results, for the purposes of this research, we define ‘stress’ as the level of HPA activity, a ‘stress response’ as a measurable increase in HPA activation, quantified by an appropriate physiological metric and a ‘stressor’ as a stimulus that increases HPA axis activity (Johnstone *et al.,* 2012).

It is important to consider the activity of the HPA axis of animals in conservation breeding programs because they are exposed to unique stressors such as transportation and relocation to a novel environment (Teixeira *et al.,* 2007; Gelling *et al.,* 2010; Taylor *et al.,* 2017; Fischer and Romero, 2019). Moreover, because animals are maintained in both *in* and *ex situ* environments and can be moved between these environments, they are exposed to different stressors that may increase HPA axis activity. Chronic stress in released animals can negatively impact their behaviour and survival post-release (Kelly *et al.,* 2008; Batson *et al.,* 2017; Lopes *et al.,* 2017). This can be mitigated by adapting management of conservation programs, such as integrating soft-release protocols (Letty *et al.,* 2000; Reading *et al.,* 2013; Tetzlaff *et al.,* 2019).

The Vancouver Island marmot (VIM) is a social, burrowing rodent, endemic to Vancouver Island, British Columbia, and currently has a conservation breeding program in place (COSEWIC, 2019). Although the VIM population grew throughout the 1980s due to increased reproduction in newly logged habitats, over-predation in these habitats led to dramatic declines throughout the 1990s and early 2000s (COSEWIC, 2019). In response to low population numbers, the recovery plan for the VIM was updated to include supplementation with VIMs from a conservation breeding program (McAdie, 2004). Consequently, four facilities began breeding VIMs with the intent of restoring populations in the wild. Five hundred forty VIMs have been released from the conservation breeding program to Vancouver Island since 2003, and the current population is estimated to be just >200 individuals at 20 colonies (Jackson *et al.,* 2020). Although there has been population growth, the wild population is not yet considered stable and is still highly dependent on supplementation from the conservation breeding program (COSEWIC, 2019).

Hair cortisol was found to be significantly elevated in newly released VIMs compared with wild or captive conspecifics, suggesting there is variation in HPA axis activity in individuals in the VIM conservation breeding program (Acker, 2017). However, there has not yet been a published study using any other metric of stress for this species. The relative proportion of neutrophils to lymphocytes in the blood, known as the N:L ratio (Davis *et al.,* 2008) is becoming more prevalent as an index of stress. The applications of the N:L ratio to stress physiology have been reviewed elsewhere (Davis *et al.,* 2008; Davis and Maney, 2018), and an increase in the N:L ratio in response to a stressor has been documented in a variety of taxa in both laboratory and field-based experiments (Vera *et al.*, 2013; Swan and Hickman, 2014; Książek *et al.,* 2017; Carbillet *et al.,* 2019). Investigating the N:L ratio in VIMs would provide a more comprehensive picture of HPA axis activity in addition to hair cortisol levels and would provide an opportunity for comparison between the metrics because cortisol levels and the N:L ratio are not always correlated (Davis and Maney, 2018).

Moreover, a key advantage to using the N:L ratio as a metric of stress is that for most species, an increase in the N:L ratio occurs hours to days after exposure to a stressor (Davis *et al.,* 2008; Martin, 2009; Breuner *et al.,* 2013). This circumvents issues of confounding stress measurements from trapping, handling, and sampling procedures, which is common when using GCs (Davis and Maney, 2018). Although the exact timing of the N:L ratio response is unknown for VIMs, the N:L ratio was not significantly elevated in Richardson ground squirrels (*Spermophilus richardsonii*) 4 hours post-capture (Delehanty and Boonstra, 2009). Blood samples are routinely taken from VIMs during medical procedures such as surgeries to implant a small tracker pre-release, or annual medical check-ups and similar methods have been used to evaluate the N:L ratio in other marmot species (Kroeger *et al.,* 2021; Uchida *et al.* 2022). Moreover, the N:L ratio can be used as an indicator of chronic stress because the metric is slower acting and remains elevated for longer than the GC response (Davis *et al.,* 2008).

The purpose of this study was therefore to investigate the N:L ratio in VIMs in the conservation breeding program. This study capitalized on amassed blood samples that have been routinely collected from individuals in the program since 1992. The objectives of the study were 2-fold: first, to compare the N:L ratio of individuals in different environments in the program including captive, pre-release, post-release and wild populations. Moreover, comparisons were made within these populations to determine if there was a difference among managed care facilities and wild meta-populations. The second objective was to determine what factors are contributing to variation in the N:L ratio in the four aforementioned populations. We predicted that: 1) the N:L ratio would vary between target populations such that captive individuals would have the highest N:L ratio as a result of artificial housing conditions, handling and irregular social structures; 2) there would not be a pronounced difference in the N:L ratio among captive facilities because animal care protocols are standardized and 3) factors contributing to variation in the N:L ratio for each population would vary due to the environment (e.g. captive vs wild).

## Methods

### Sampling populations and locations

VIMs were assigned to the captive population if they are born in captivity, or any wild VIM taken into captivity for breeding purposes that have remained in captivity for longer than one hibernation period (McAdie, 2018). The captive population of VIMs is currently housed at three sites: The Toronto Zoo (TZ), Calgary Zoo (CZ), and the Tony Barrett Mt. Washington Marmot Recovery Centre (TBMWMRC)(Carnio 2019). VIMs were also bred at the Mountain View Conservation and Breeding Society (MVF) from 2000 until 2014. Blood samples were routinely collected from captive VIMs as part of annual health screens, pre-surgical or pre-shipment health screens.

VIMs were assigned to the release population if they were born in captivity and released, or if they were born in the wild but spent at least one hibernation period in captivity and were then released back to the wild. This population was further subdivided into the pre-release and post-release populations, where pre-release were VIMs temporarily housed at the TBMWMRC before release. This population was treated as independent from the captive population because samples were taken after being relocated to the TBMWMRC and for different reasons, implanting the transmitter and a pre-release health screen. Pre-release individuals could have been housed at any of the other care facilities before release and may have only been housed at the TBMWMRC for a brief quarantine period. The post-release population were released VIMs that were recaptured after release and sampled opportunistically or to replace the transmitter.

VIMs in the wild population were individuals born in the wild and had not spent extended time in captivity (McAdie, 2018). VIMs are being released to a number of sites within two primary areas—referred to as meta-populations—on Vancouver Island: Strathcona Provincial Park and Nanaimo Lakes (Aaltonen *et al.,* 2009; Vancouver Island Marmot Recovery Team, 2017). This analysis focused on the meta-population scale. Blood samples were opportunistically obtained from wild VIMs during recapture events or when transmitters were implanted or replaced. These blood draws typically happened in the field, and the individual was not taken into captivity.

### Blood sample collection, storage and analysis

All samples used in this analysis were from clinically healthy marmots, which were defined as ‘marmots that do not display cardiovascular, respiratory, musculoskeletal, nervous, integumentary or urogenital abnormalities after an examination by a veterinarian’ (McAdie, 2018). As outlined by McAdie (2018), whole blood samples were collected from the cephalic, saphenous, femoral or tarsal veins after immobilization using an intramuscular injection of ketamine hydrochloride and midazolam hydrochloride (10 and 0.25 mg/kg, respectively). A portion of the blood collected was used to make two blood smears, which were stained and dried at room temperature (or ambient temperature in field conditions), and stored in cool, dry, dark conditions (McAdie, 2018).

These blood smears were previously used to establish 16 reference values for the species including neutrophils, lymphocytes, monocytes, eosinophils and basophils (See: McAdie, 2018). The N:L ratio was calculated using the total neutrophil count (segmented and banded), divided by the lymphocyte count (Hickman, 2017). For 50 samples analysed in 2020, the N:L ratio was calculated using the percentage neutrophil count divided by the percentage lymphocyte count.

### Statistical analysis

All statistical analysis was conducted in R (R Core Team 2020). To compare the N:L ratio among target management groups, linear mixed models were created using the *lme4* package (Version 1.1.23; Bates *et al.,* 2015). The first model (referred to as the population model) compared the N:L ratio among the four populations: captive, pre-release, post-release and wild. Two other models were created to compare individuals within these populations as follows: a model using only captive individuals was used to compare the four captive facilities and a model using only wild individuals was used to compare the two meta-populations.

For each of the aforementioned models, the N:L ratio was log transformed to achieve normality and used as the response variable. Sex and month of sampling were accounted for in each model as fixed effects, and year was included as a random effect in each model to account for unequal sampling effort among years. Individual was included as a random effect in each model save the model of wild individuals to account for multiple samples from the same individuals. In the model of wild individuals, including this random effect led to overfitting due to limited repeat samples from the same individual, so one sample per individual was selected at random. Age was evaluated as a fixed effect in each model except for the population model because of unequal variation in the ages among the population. For example, most of the pre-release VIMs were aged <1 year because this is the age they are typically released from managed care (Lloyd *et al*. 2019), whereas post-release VIMs were largely aged between 2 and 3 years because this is when the transmitter is replaced. Therefore, including age in this model led to overfitting, and it was removed. Finally, all blood samples were analysed at one of three laboratories for the sampling period: IDEXX, Central Veterinary Lab (CVL) and TZ Lab. To account for differences in sampling technique and experience that may confound cell counts, laboratory was included as a fixed effect. However, in the model of captive individuals, this led to collinearity because each facility used the same laboratory to analyse their samples. Therefore, the effect laboratory was removed from this model. See Table 1 for the complete models.

**Table 1:** Summary of the models used for comparisons of the N:L ratio for this analysis. For each model, the N:L ratio was log transformed and used as the response variable

Model name	Target comparison	Predictors
Population	Populations	Sex + month + laboratory + (1|individual) + (1|y)
Captive	Care facilities	Sex + age + month + (1|individual) + (1|y)
Wild	Wild meta-populations	Sex + age + month + laboratory + (1|y)

Model visualization was performed using the *visreg* package (version 2.6.1; Breheny and Burchett, 2017). To determine significant differences between the target categories displayed on the figures, an analysis of variance (ANOVA) test was conducted on the model. The *emmeans* package (version 1.5.5; Lenth *et al.,* 2021) was used to calculate the estimated marginal means for the target effect (e.g. the population, the care facility, etc.) and contrasted using a Tukey honest significant difference test.

To compare the sources of variation in the N:L ratio among populations, a linear mixed model was created for each of the four populations. Each model used the log transformed N:L ratio as the response variable. The fixed effects in the model included sex, age and the month the sample was taken, and a random effect of year and a random effect of individual. Numeric predictors were scaled for comparison. For the model of the wild population and the post-release population, including individual as a random effect led to overfitting in the model due to limited repeat samples from the same individual. However, a comparison between these models, and models that experimentally removed the random effect of individual by randomly selecting one sample per individual yielded no significant differences in outcome in terms of magnitude of effect or significance (see Appendix; Supplementary Fig. S1). Therefore, the random effect of individual was kept in the wild and post-release population models to compare with the other two populations. Models were visualized and compared using the *jtools* package (version 2.1.0; Long, 2020).

## Results

A total of 1237 blood samples were analysed from 509 marmots, collected between 1992 and 2020. The number of samples per individual ranged from 1 to 15, with a mean of 2.43 ± 2.02 SD samples per individual. Of these 1237 samples, 739 were from captive individuals, 348 were from pre-release individuals, 24 were from post-release individuals and 126 were from wild individuals. All samples were collected in the active season between April and October, with most samples collected in either September or October (*n* = 628) because this is when annual examinations typically happen for individuals in captivity. Five hundred ninety-six samples were collected between June and August, 11 samples were collected in May and 2 samples were collected in April.

### Comparing populations

**Figure 1:**
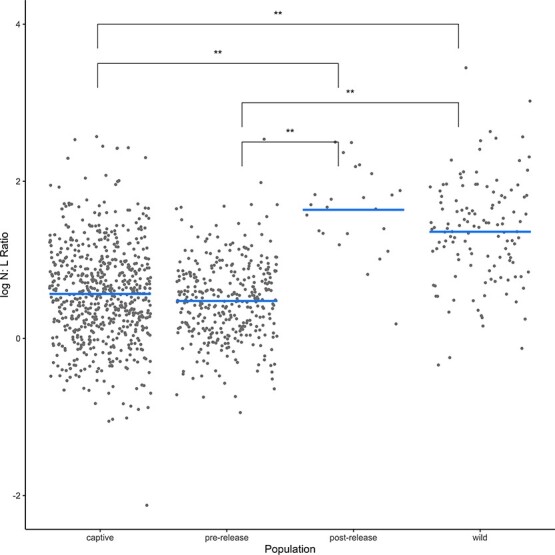
Partial residuals of log N:L ratio among each population. Captive samples (*n* = 739) are collected for annual examinations, pre-release samples (*n* = 348) are collected for implant surgeries or pre-release examinations, post-release samples (*n* = 24) are collected for replacement of transmitters and wild samples (*n* = 126) are collected opportunistically for replacement of transmitters, routine field processing and trapping for captivity.

There were significant differences in the N:L ratio among the populations [*F*(3, 1097.94) = 54.81, *P* < 0.001]. A Tukey significant difference test of the estimated marginal means yielded significant differences in the N:L ratio between *ex situ* populations (captive and pre-release) and *in situ* populations (post-release and wild). The N:L ratio for *ex situ* populations was significantly lower than post-release and wild (*P* < 0.001, respectively, Fig. 1). There was no significant difference between the captive and pre-release populations, nor between the post-release and wild populations.

### Comparing care facilities

**Figure 2:**
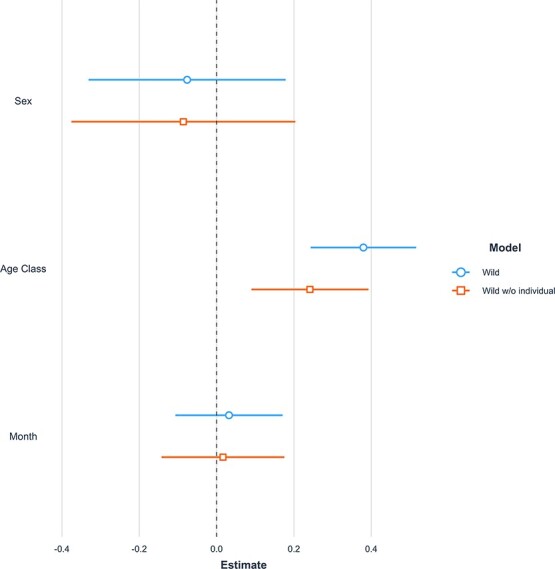
Comparison of partial residuals of log N:L ratio among care facilities. This consisted of CZ (n = 228), MVF (n = 140), TBMWMRC (n = 227) and TZ (n = 141).

A total of 736 samples from 300 captive individuals were analysed. This consisted of 228 samples from CZ, 140 samples from MVF, 227 samples from the TBMWMRC and 141 samples from TZ. There were significant differences in the N:L ratio among the facilities [*F*(3, 486.21) = 19.06, *P* < 0.001]. *Post hoc* tests suggest the N:L ratio was significantly higher at MVF and CZ compared with the TBMWMRC and TZ (*P* < 0.01; Fig. 2). There was no significant difference in N:L ratio between individuals at TZ and the TBMWMRC or between MVF and CZ.

### Comparing wild populations

**Figure 3:**
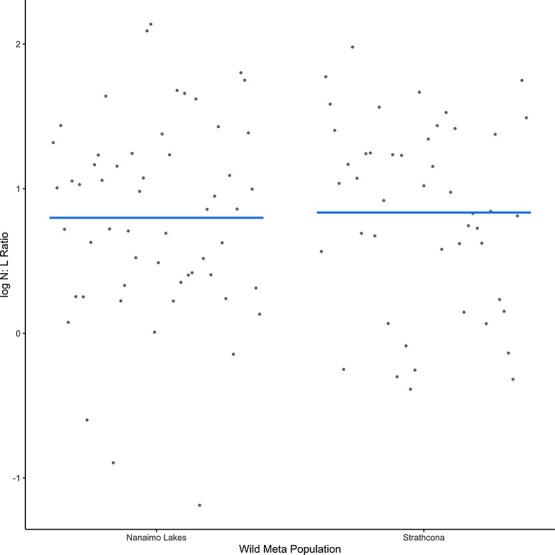
Comparison of partial residuals for log N:L ratio between meta-populations.

A total of 105 samples from wild individuals were analysed. This consisted of 58 samples from Nanaimo Lakes and 47 samples from Strathcona. There were no significant differences in the N:L ratio between the two meta-populations (Fig. 3).

### Sources of variation in the N:L ratio for the populations

**Figure 4:**
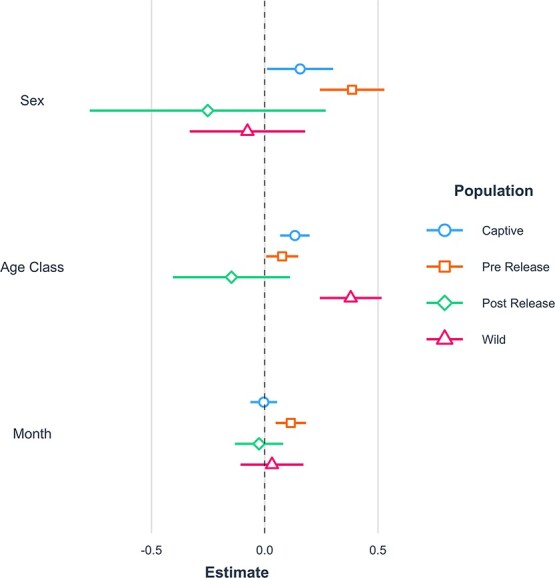
Comparison of estimates for three predictors for population models.

The effect of age, sex and the month the sample was taken on the N:L ratio was compared among each of the four populations. The relative impact of each predictor was assessed through a comparison of the effect sizes ($\beta$). Sex was a significant predictor in captive and pre-release models (captive: *P* = 0.04, $\beta$ = 0.16 and pre-release: *P* < 0.001, $\beta$ = 0.39; Fig. 4); however, not in the wild or post-release models. Males in the captive and pre-release populations had higher N:L ratios than females. Age was a significant predictor for all populations except for post-release and had the strongest positive effect in the wild population (*P* < 0.001, $\beta$ = 0.46; Fig. 4). The post-release population included one sample that seemed as an outlier despite falling within 3 SD of the mean N:L ratio for that group. Inclusion of this data point led to a negative effect of age on the N:L ratio, but removal of the data point resulted in a significant positive effect of age on the N:L ratio, with a relatively strong effect size (*P* < 0.001, $\beta$ = 0.57). The latter result was consistent with the positive relationship between age and the N:L ratio in the other VIM groups. There was no obvious biological reason why the data point was an outlier. Finally, the month the sample was taken was only significant in the pre-release population, such that samples taken later in the year have a higher N:L ratio (*P* < 0.001, $\beta$= 0.13).

## Discussion

This study used the N:L ratio to compare physiology of VIMs among and within different managed groups in the conservation breeding and release program and assessed factors contributing to variation in this metric. The results show there are differences in the N:L ratio between *in situ* and *ex situ* groups. VIMs *in situ,* either wild-born or post-release, have a significantly higher N:L ratio than those *ex situ*. There was no difference in the N:L ratio between the captive and pre-release groups, nor between the wild and post-release groups, which indicates they could be considered together for future analysis. Moreover, the N:L ratio differed based on which care facility the VIMs were housed in within the captive population. Finally, an analysis of the relative effect of three predictors known to affect HPA axis activity indicated that there is a difference in what influences the N:L ratio in VIMs based on which population they are in. These results support the consideration of conservation physiology in this breeding program because the physiology of VIMs is not the same between *in* and *ex situ* environments, nor among care facilities.

### N:L ratio among target comparisons

It was unexpected that the N:L ratio of VIMs *ex situ* was lower than *in situ*. Other studies have found stress to be higher in captive animals compared with their wild conspecifics. This was the case for African wild dogs (*Lycaon pictus*; Van der Weyde *et al.,* 2016), Canada lynx (*Lynx canadensis*; Fanson *et al.,* 2012)) and cheetahs (*Acinonyx jubatus*; Terio *et al.,* 2004). This has been attributed to stressors such as visitor traffic, artificial housing conditions, and interactions through zoo husbandry practices (Terio *et al.,* 2004; Morgan and Tromborg, 2007; Fanson *et al.,* 2012; Van der Weyde *et al.,* 2016). Although VIMs in managed care are in artificial conditions compared with the wild environment, they are housed separately from the public viewing collection at both zoos. This may be contributing to the lower-than-expected N:L ratio in this study.

There are two hypotheses for the difference in N:L ratio between *in* and *ex situ* VIM populations. The first hypothesis, that *in situ* VIMs are experiencing an increased N:L ratio as a result of a more physiologically challenging environment, is strengthened by studies that demonstrate that HPA axis activity can increase with concurrent stressors, although this effect is variable in some individuals (Blanchard *et al.,* 1998; Kim *et al.,* 2013; Spencer and Deak, 2017). It is possible that exposure to multiple, unpredictable stressors for VIMs in the wild such as contact with predators, variation in weather, and social competition is resulting in elevated HPA axis activity, and thus an increased N:L ratio. It is unlikely that this increase in the N:L ratio for *in situ* VIMs was the result of an immune response because monocytes and eosinophils were not found to be significantly elevated between groups in previous analysis (McAdie, 2018). Because there are now two studies using two different stress metrics (hair cortisol and the N:L ratio) that show elevation in *in situ* individuals, it supports the theory that the wild is a more challenging environment than managed care for this species (Acker, 2017).

The second hypothesis that may explain the difference in N:L ratio involves acclimation of HPA axis activity because of repeated stressors for *ex situ* VIMs. Acclimation occurs when repeated stressors suppress HPA axis activity and can result in lower GC release (Romero, 2004; Rich and Romero, 2005; Spencer and Deak, 2017). Despite being housed apart from public zoo visitors, the managed care of VIMs still involves a regimented schedule (e.g. daily feedings, cleanings, annual examinations, etc.) Therefore, it is possible that VIMs *ex situ* are exhibiting a lower N:L ratio than their wild conspecifics as a result of a suppressed HPA axis (Romero, 2004). Although it is impossible to say if this is happening using the results of the present study, this seems unlikely given that VIMs *ex situ* are typically in good body condition, do not experience a compromised immune system (McAdie, 2018) and were shown to have similar breeding rates to wild individuals (Bryant, 2005), which can all be indirect indicators of chronic stress. However, because this cannot be ruled out entirely, it is encouraging that our results showed no significant difference in the N:L ratio between post-release and wild individuals, indicating they are in a similar physiological state with respect to the N:L ratio. This implies that if there is dysregulation of the HPA axis as a result of managed care, these effects are not lasting. However, future studies should challenge the HPA axis and focus on the ability of VIMs to cope with a stressor to confirm if there are fitness consequences as a result of the observed differences.

The N:L ratio did vary based on the care facility. Overall, VIMs housed at the TBMWMRC had the lowest N:L ratio, which was significantly lower than VIMs at CZ and MVF, but not TZ. A study using Asian elephants (*Elephas maximus*) found captive elephants with access to outdoor enclosures in their natural habitats had significantly lower stress (Kumar *et al.,* 2019). This suggests that access to outdoor space within the natural habitat at the TBMWMRC may have contributed to a lower N:L ratio. The N:L ratio of VIMs housed at MVF was significantly higher than that at the TBMWMRC. There are notable differences in elevation between the TBMWMRC (1244 metres above sea level [m.a.s.l.]) and MVF (25 m.a.s.l), as well as differences in the housing infrastructure and husbandry that VIMs received at these facilities that may be contributing to these differences (M. McAdie pers.comm 2021). The N:L ratio was likewise significantly higher at CZ compared with the TBMWMRC and TZ. These results together suggest that a combination of environmental factors, including access to outdoor space in the natural habitat, housing infrastructure, as well aspects of management within the facility such as daily housing and husbandry are contributing to the N:L ratio of VIMs in managed care. In addition, each care facility used a different laboratory to analyse their samples. To further investigate possible confounding variables from sampling techniques and analysis, samples from all facilities should be analysed at one laboratory.

Habitat and geographic range may also influence stress, as demonstrated by a study of free-ranging roe deer (*Capreolus capreolus*), which found significant variation in the N:L in three different habitats (Carbillet *et al.,* 2019). However, the N:L ratio was not significantly different for VIMs in the two meta-populations in this analysis. The similarity in N:L ratio between the two meta-populations may be due to the types of stressors and environmental characteristics experienced in the wild (Romero *et al.,* 2008; Edwards *et al.,* 2016; Ozella *et al.,* 2017; de Bruijn and Romero, 2018) and the proximities of the two meta-populations (<100 km; Aaltonen *et al.,* 2009). To fully understand how or if the stress of wild VIMs is different based on the colony location, more samples would need to be taken from each site.

### Sources of variation in N:L ratio

The relative contribution of age, sex and the month of sampling to variation in the N:L ratio was different for the four populations of interest. Age was a significant predictor of the N:L ratio for all populations except for post-release, most likely due to a smaller sample size (*n* = 24) because one sample drove the relationship in the opposite direction. However, when this sample was removed, the N:L ratio did increase significantly with age in this population. Overall, the effect of age was the largest for *in situ* VIMs. Other studies have that found HPA axis activity increases with age when measured using GCs (Reeder and Kramer, 2005) and the N:L ratio (Watson *et al.,* 2016; Carbillet *et al.,* 2019; Uchida *et al.,* 2021). Animals in managed care may have specialized treatments to mediate age-related physiological deteriorations and diseases. For example, a survey of 63 zoos housing African and Asian elephants found that ‘managed time’ (i.e. time spent engaged with staff for training, feeding, exercise, etc.) increased by 20.2% with each year of age, primarily to combat health risks associated with older individuals (Greco *et al.,* 2016). The specialized care of older captive VIMs that experience health complications may mediate the effect of age on the N:L ratio. Moreover, older VIMs in the wild are subjected to increased social and territorial pressure from younger individuals, which could likewise contribute to a larger effect of age in that population (M. McAdie pers. comm. 2021; Uchida *et al.,* 2021). Although it is unclear if there is a relationship among stress, age and fitness, a study investigating age-specific survival rates of released VIMs found apparent survival of older age classes (>3 years) was lower than in closely related species (Aaltonen *et al.,* 2009). Further investigation into the mechanism of age-related stress responses in VIMs is needed to determine if this survival rate was impacted by stress. Because VIMs are an endangered species, sample sizes will be small; however, this makes the trend for *in-situ* individuals, particularly post-release VIMs, harder to evaluate. Increased sampling of post-release individuals would be beneficial to understand this trend for this managed group.

There were notable differences in the effect of sex on the N:L ratio in different populations. Sex was a significant predictor of the N:L ratio in *ex situ* populations of VIMs, but not in post-release or wild. Specifically, males *ex situ* exhibited a higher N:L ratio than females, although overall, this was still lower than for *in situ* males. In wild mammals, there is a general consensus that HPA axis activity (measured with GCs) is higher for females than males (Reeder and Kramer, 2005). This is attributed to high energetic costs associated with reproduction. There is less of a consensus about the N:L ratio between sexes particularly in the context of stress. Studies using ground squirrel species have found no differences in the N:L ratio between sexes (Wenberg *et al.,* 1973; Barker and Boonstra, 2005; Desantis *et al.,* 2016), and similarly no difference in rats (Hickman, 2017). However, a study using the N:L ratio in roe deer did find the N:L ratio to be higher in males (Carbillet *et al.,* 2019).

The structure of social animals, particularly rank, can affect HPA axis activity (Creel *et al.,* 2013), and may explain some of the variation in N:L ratio for VIMs in managed care. In the wild, VIMs live socially in colonies, often with more than one family *(*Aaltonen *et al.,* 2009). In managed care, VIMs are usually housed in breeding pairs. They are maintained in proximity to each other and can likely see, hear or smell other males without the ability to establish and maintain social rankings. A study on the alpine marmot (*Marmota marmota*) found that the N:L ratio was higher in dominant males than in subordinate but sexually mature males (Cohas *et al.,* 2018). Because males in breeding pairs have access to females and can exhibit breeding behaviors, and thus are dominant, it may be the case their N:L ratio is similarly elevated and so, overall male N:L ratios are elevated in managed care because of the absence of subordinate males. However, a study in yellow-bellied marmots found the N:L ratio decreased with rank (Uchida *et al.,* 2021). Although it seems that the N:L ratio is affected by rank or social dominance in marmots, there is no consensus as to which direction. A study examining the N:L ratio in breeding versus non-breeding males, compared with wild conspecifics, would be needed to confirm why the N:L ratio seems to be higher in *ex situ* males.

Although other research has found that hematological parameters and basal GCs change seasonally in a variety of species (Boonstra *et al.,* 2001; Vera *et al.,* 2011), in this study, the month the sample was taken was only significant in the model of pre-release VIMs, and the overall effect in the model was quite small. This is not to say seasonal variation is unimportant; however, the lack of significance of this predictor in the current analysis is likely due to the restricted range of the sampling schedule. In each population, samples are taken approximately the same time and all within the active season. There are no samples taken during hibernation due to possible complications with arousal, concerns with diminished platelets, and hemostasis (McAdie, 2018). In addition, there are no samples collected during the breeding season. Therefore, it is possible that there were not enough samples collected in the months leading into hibernation (November) and emergence (April–May), nor breeding, to capture this seasonal variation.

## Conclusion

This study has shown not only that there are significant differences the N:L ratio of VIMs in different managed groups in the program, but there are also significant differences in factors contributing to variation in this metric. Because the apparent survival of captive-bred VIMs is lower than wild conspecifics (Lloyd *et al.,* 2018), it is important to consider physiological differences between *in situ* and *ex situ* individuals to maximize our understanding of fitness for all individuals in the population. Because this study was a retrospective analysis of data and there are inherent limitations of these data (i.e. small sample sizes for certain groups), it is important for further experimental evaluation of these trends to determine their causes and whether it is necessary to mitigate their effects.

## Funding

This work was supported by the ReNew Zoo CREATE grant provided by The Natural Sciences and Engineering Research Council of Canada. [grant number 481954–2016].

## Author Contributions

S.F., M.M., G.M. and A.S.H. all contributed to the study design. S.F. performed the statistical analysis and wrote the manuscript. M.M. provided data used in the analysis. G.M., M.M. and A.S.H. provided edits to the manuscript.

## Conflict of Interest

The authors have no conflicts of interest to declare.

## Data Availability

Data were provided with special permissions. Given the sensitivity of the data, any data or research requests should be discussed with the Marmot Recovery Foundation (info@marmots.org).

## Supplementary Material

Web_Material_coad041
