# Supplementary material for: Assessing stress physiology within a conservation breeding program for an endangered species
Source: Conserv Physiol. 2023 Jun 9;11(1):coad041. doi: 10.1093/conphys/coad041 (PMC10660376; doi:10.1093/conphys/coad041)
Supplement: Web_Material_coad041 [file web_material_coad041.pdf]

Supplementary Data

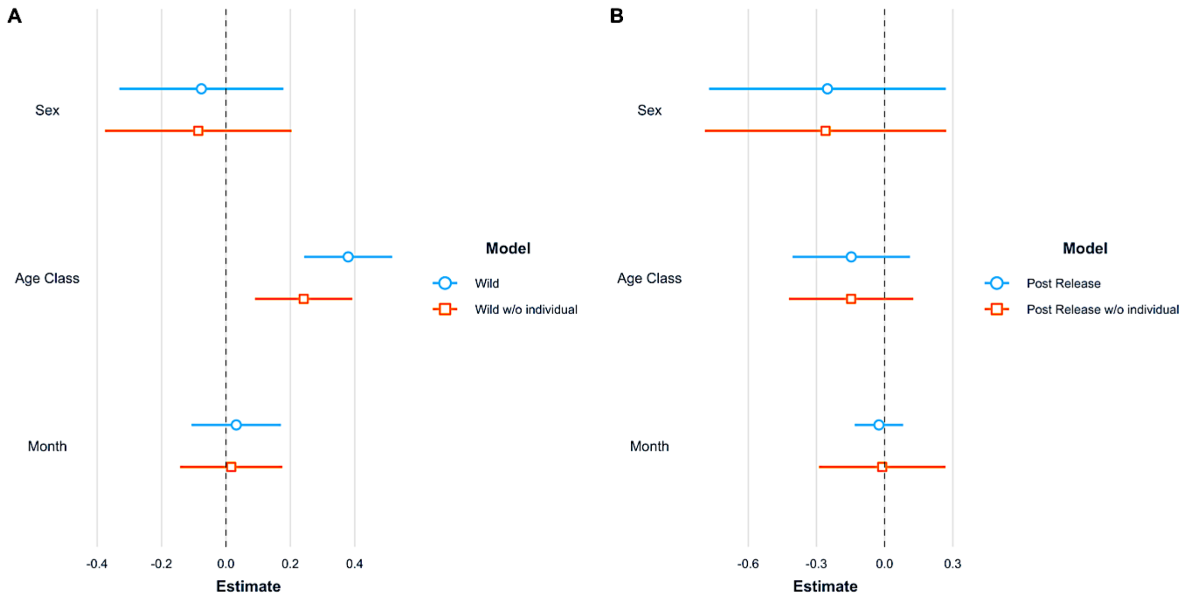

Figure A1. Comparison of estimates for the wild model (A) and post-release (B) with and without the random effect of individual.
